# Hepatitis B and C Co-Infection among HIV-Infected Adults while on Antiretroviral Treatment: Long-Term Survival, CD4 Cell Count Recovery and Antiretroviral Toxicity in Cambodia

**DOI:** 10.1371/journal.pone.0088552

**Published:** 2014-02-12

**Authors:** Johan van Griensven, Lay Phirum, Kimcheng Choun, Sopheak Thai, Anja De Weggheleire, Lutgarde Lynen

**Affiliations:** 1 Sihanouk Hospital Center of HOPE, Phnom Penh, Cambodia; 2 Institute of Tropical Medicine, Antwerp, Belgium; Centers for Disease Control and Prevention, United States of America

## Abstract

**Background:**

Despite the high burden, there is a dearth of (long-term) outcome data of hepatitis B virus (HBV) and hepatitis C virus (HCV) co-infected patients receiving antiretroviral treatment (ART) in a clinical setting in resource-constrained settings, particularly from Asia.

**Methods:**

We conducted a retrospective cohort study including all adults initiating standard ART (non-tenofovir-based) between 03/2003 and 09/2012. HBV infection was diagnosed by HBV surface antigen detection. HCV diagnosis relied on antibody detection. The independent effect of HBV and HCV on long-term (≥5 years) ART response in terms of mortality (using Cox regression), severe livertoxicity (using logistic regression) and CD4 count increase (using mixed-effects modelling) was determined.

**Results:**

A total of 3089 adults were included (median age: 35 years (interquartile range 30–41); 46% male), of whom 341 (11.0%) were co-infected with HBV and 163 (5.3%) with HCV. Over a median ART follow-up time of 4.3 years, 240 individuals died. Mortality was 1.6 higher for HBV co-infection in adjusted analysis (*P* = 0.010). After the first year of ART, the independent mortality risk was 3-fold increased in HCV co-infection (*P* = 0.002). A total of 180 (5.8%) individuals discontinued efavirenz or nevirapine due to severe livertoxicity, with an independently increased risk for HBV (hazard ratio (HR) 2.3; *P*<0.001) and HCV (HR 2.8; *P*<0.001). CD4 recovery was lower in both HBV and HCV co-infection but only statistically significant for HBV (*P*<0.001).

**Discussion:**

HBV and HCV co-infection was associated with worse ART outcomes. The effect of early ART initiation and providing effective treatment for hepatitis co-infection should be explored.

## Background

The roll out of antiretroviral therapy (ART) has had a major impact on HIV-associated mortality in low and middle income countries, and HIV is now gradually becoming a chronic condition. Co-morbidities like hepatitis B virus (HBV) and hepatitis C virus (HCV) infection currently pose major clinical and public health challenges [1], [2]. Chronic HBV infection affects over 240 million people worldwide; there are an estimated 150 million cases of HCV infection [3]–[5]. At the global level, Asia carries the largest burden, and also hosts a total of 5 million HIV infected individuals [1], [6], [7]. Both viral hepatitis infections are associated with more rapid progression of liver fibrosis in the event of HIV co-infection and liver pathology has now been identified as a leading causes of death in high-income countries [1], [8], [9]. In these countries, HBV and HCV treatment, following specific indications, is part of the HIV care package.

In most low and middle income countries, management and monitoring of HBV and HCV is not integrated in public ART programs, although some antiretrovirals do exert activity against HBV [6], [10]. In this setting, HBV and HCV infection remain typically undiagnosed and co-infected individuals only receive ART [6]. Although ART-mediated CD4 recovery and reducing HIV-related immune activation and inflammation could contribute to HBV and HCV control, enhanced ART-related toxicity is obviously a concern [10]–[14]. There is however a dearth of outcome data of HCV and HBV co-infected patients receiving ART in a clinical setting in resource-constrained settings. Such information is essential to position the need to prioritize the provision of treatment for hepatitis co-infection in low and middle income countries and to develop evidence-based guidelines and policies. This is particularly important since HBV and HCV treatment currently remains prohibitively expensive, is associated with substantial toxicity or relies on expensive laboratory tests [2], [5], [6]. It is also not clear whether in these settings, co-infected individuals should be prioritized for early ART initiation, as is currently recommended in European and American guidelines [15]–[17].

Although Asia carries a large burden of HBV and HCV co-infection, studies from this continent, especially from low income countries, are proportionally scarce and have yielded conflicting findings [18]–[22]. For instance, one recent report on HCV/HIV co-infection from Thailand found no substantial effect of HCV on morbidity, mortality or treatment responses to ART, arguing against prioritizing HCV treatment within HIV treatment programs [19]. However, reported studies generally suffered from one or more limitations including small sample size, short follow-up or were conducted prior to the ART scaling-up. Based on carefully collected program data over a period of ten years, we report on the prevalence of HBV and HCV co-infection, and the effect on ART response in term of CD4 cell count recovery, all-cause mortality and ART-related drug toxicity in adult patients on ART in Cambodia.

## Methods

### Study design and study population

We conducted a retrospective cohort study at the Sihanouk-Hospital-Center-of-Hope (SHCH) in Phnom Penh, Cambodia. This non-governmental hospital provides comprehensive HIV care free of charge since March 2003, as part of the national ART program. All adult HIV-infected patients initiating standard (stavudine or zidovudine-based) first line ART at the hospital between March 2003 and September 2012 were included. Those started on alternative ART regimens and those missing any of the hepatitis tests were excluded.

### Antiretroviral treatment initiation and monitoring

Indications for ART initiation followed WHO recommendations: all patients with WHO stage IV, WHO stage III with CD4 cell count<350 cells/μL or with CD4 cell count<200 cells/μL were eligible for ART. From June 2010 on, a CD4 threshold of<350 cells/μL was used. Standard first line treatment consisted of a generic fixed dose combination containing stavudine, lamivudine and nevirapine. In case of contraindications to stavudine or nevirapine, zidovudine or efavirenz was prescribed. For co-infected patients, guidelines recommended cautious use of nevirapine and initiation of efavirenz based on clinical and laboratory indications of liver disease.

During the pre-ART preparatory work-up, all patients received extensive counselling before ART and concurrent opportunistic infections were ruled out. Patients were seen at two and four weeks after starting ART, followed by monthly visits. After the first six months of ART, visits were scheduled less frequently (every 2–3 months) for clinically stable patients. All medical care was provided by physicians, supported by a team of nurses and adherence counsellors. At every clinical encounter, a number of key issues were systematically addressed, including the assessment of treatment response and ART-related toxicity. All toxicity events were graded using the WHO severity scale (I to IV) and recorded on standardized data collection sheets. In case of non-nucleoside reverse transcriptase inhibitor (NNRTI) associated hepatotoxicity, drug discontinuation was indicated (“treatment-limiting hepatotoxicity”) for all grade IV changes in transaminases (serum alanine aminotransferase (ALT)>10 times the upper limit of normal) or any symptomatic liver toxicity. Adherence assessment relied on pill counts (at every visit), and the visual analogue scale (every six months). Baseline laboratory testing included haematology, liver function tests, hepatitis B/C testing and CD4 cell count determination (FACSCount (Becton Dickinson). For HBV infection, hepatitis B surface antigen was determined. Hepatitis C diagnosis relied on antibody detection. Both tests were done using a chemiluminescence immunoassay (CIA) on a Cobas e 411 analyzer (Roche Diagnostics, Mannheim Germany) from 2009 on, and an AxSYM analyzer (Abbott laboratories, Illinois, US) before that. No additional viral hepatitis tests (antigen, serology, molecular) were available in the program. After ART initiation, a full blood count and CD4 cell count was done every six months. Liver function tests were done at month 1, 2, 3, 6 after ART initiation, followed by six-monthly measurements. A viral load test was done in case of clinical or immunological indications of treatment failure. Cotrimoxazole prophylactic treatment was given for all WHO stage II/III/IV patients and all those with a CD4 count<200 cells/μL. All patients with WHO stage IV disease or a CD4 count<100 cells/μL were started on fluconazole primary prophylaxis and were screened for cryptococcal antigenemia. Patients not presenting at their scheduled visit were contacted by phone. Those living in the neighbourhood of the hospital were visited at home. Patients not presenting at the hospital for a period of 6 months without additional information were defined lost to follow-up (LTFU). Additional program details and outcome data of the antiretroviral treatment program in SHCH have been published before [10], [11].

### Data collection and statistical analysis

Clinical and laboratory data were prospectively collected on a daily basis, using standardized data collection tools and stored in a database. All physicians were systematically trained in the use of the case definitions and patient management according to the hospital guidelines. ART-toxicity grading followed WHO recommendations. Quality control of the stored data was done at regular intervals.

Baseline patient characteristics were described and compared using χ2 or Fisher's exact tests for categorical variables and the Wilcoxon rank-sum test for continuous variables. There were three main outcomes: 1) CD4 increase over time after ART initiation; 2) time to death while on ART; 3) discontinuation of NNRTI due to toxicity (rash, livertoxicity), also defined as treatment-limiting (severe) toxicity. The main exposure was HCV (positive serology at baseline) and HBV co-infection (detectable HBV surface antigen at baseline).

#### CD4 count increase

In descriptive analysis, the mean CD4 count and 95% CI was calculated at different time points, up to five years after ART initiation. The independent effect of hepatitis co-infection on the change in CD4 count after ART initiation was estimated using mixed effects modeling, as previously described in detail [23]. In secondary analysis, time to achieving a CD4 count>350 cells after ART initiation was taken as outcome using Cox regression modeling.

#### Survival

Person-time at risk was calculated, starting from the date of ART initiation up to either the date of death, date of last visit for those LTFU or transferred-out, and 30 September 2013 for the remainder. Cumulative mortality was calculated using Kaplan-Meier methods. To allow the effect of hepatitis co-infection to vary over time, we constructed two separate Cox proportional hazard models with follow-up time split at one year of ART. The proportional hazard assumption was assessed graphically and tested formally using Schoenfeld residuals. In secondary analysis, the on treatment CD4 count was additionally included to control for differences in CD4 count recovery.

#### ART toxicity

The overall change in serum alanine aminotransferase (ALT) within the first year after ART initiation was visualized using a nonparametric called LOWESS smoothing (for locally weighted scatterplot smoothing, ‘lowess’ command in STATA). This provides a representative smooth curve through data using robust local regression. The independent association between hepatitis co-infection and severe NNRTI-related toxicity (hepatotoxicity or skin rash) during the first year of ART was determined using logistic regression. In secondary analysis, liver toxicity (based on ALT levels) of WHO grade II or higher while on ART was taken as outcome.

In all analyses, interaction between HBV and HCV co-infection was assessed. Collinearity was evaluated by calculating the variance inflation factors. The functional form for continuous data was determined using the mfp (multivariable fractional polynomial models) command in STATA.

In all multivariable models, potential confounding factors were considered *a priori* for inclusion based on evidence from published studies in this and other ART programs. Details of the included variables can be found in the legend of the tables. Data were analyzed using STATA version 11 (STATACorp LP, College Station, United States of America). The level of significance was set at *P*<0.05.

### Ethical issues

Since the launch of the HIV care program, clinical data have been routinely collected for purposes of program monitoring and evaluation, and research activities. Patients were requested to give informed consent to store and use the data. No linkage of these data with other sources was done. The data collection and informed consent procedure were approved by the institutional review board of the SHCH and Institute of Tropical Medicine, Antwerp, Belgium. No patient identifiers were included in the dataset used for this analysis.

## Results

### Patient characteristics

Between March 2003 and September 2012, 3466 adults initiated standard first line ART. Of these, 377 had no hepatitis testing performed and were excluded. Of the remaining 3089 patients included in the analysis, 46% were male. The median age was 35 (interquartile range (IQR) 30–41) years. The median follow-up time on ART was 4.3 (IQR 2.1–6.7) years. There were 341 (11.0%) individuals with HBV co-infection, and 163 (5.3%) with HCV co-infection. There were only twelve individuals with both HBV and HCV co-infection. The baseline characteristics of the different groups are shown in Table 1. Of the 341 HBV co-infected individuals, 77 (22.6%) were prescribed tenofovir after a median time of two years, predominantly due to intolerance to stavudine or zidovudine. Two HBV co-infected cases were started on tenofovir due to persistently elevated ALT and/or signs of livertoxicity.

**Table 1:** Baseline and clinical characteristics of adults initiating ART according to hepatitis B and hepatitis C status in Phnom Penh, Cambodia 2003–2012 (N = 3089).

	Hepatitis B(−) and Hepatitis C(−)	Hepatitis B(+)	Hepatitis C(+)
Total^a^	2597	341	163
Sex			
Male	1166 (44.9)	198 (58.1)	74 (45.4)
Female	1431 (55.1)	143 (41.9)	89 (54.6)
Age, years; median (IQR)	34 (29–40)	34 (30–39)	41 (35–47)
≤30; n (%)	801(30.8)	103 (30.2)	25 (15.3)
31–40; n (%)	1168 (50.0)	166 (48.7)	54 (33.1)
41–50; n (%)	473 (18.2)	48 (14.1)	57(35.0)
>50; n (%)	155 (6.0)	24 (7.0)	27 (16.6)
Baseline body weight, kg; median (IQR); (n = 3086)	49 (43–55)	50 (44–56)	49 (44–56)
Baseline CD4 count, cells/μL; median (IQR); (n = 3070)	104 (26–227)	80 (28–198)	103 (33–218)
Baseline ALT elevation ≥ grade 2 (n = 3032)	100/2551 (3.9)	18/330 (5.4)	8/163 (4.9)
Baseline WHO stage			
Clinical stage I/II	616 (23.7)	71 (20.8)	33 (20.2)
Clinical stage III/IV	1981 (76.3)	270 (79.2)	130 (79.8)
NNRTI at ART start			
Nevirapine	1874 (72.2)	229 (67.2)	105 (64.4)
Efavirenz	723 (27.8)	112 (32.8)	58 (35.6)
NRTI at ART start			
Stavudine	2431 (93.6)	322 (94.4)	152 (93.3)
Zidovudine	166 (6.4)	19 (5.6)	11 (6.7)
Starting tenofovir during follow-up	385 (14.8)	77 (22.6)	43 (26.4)

ART: antiretroviral treatment; NNRTI: non-nucleoside reverse transcriptase inhibitor; NRTI: nucleoside reverse transcriptase inhibitor; WHO: world health organization.

a There were 3089 individuals included in the study; since there were 12 patients with both HepB and Hep C coinfection, the total adds up to 3101.

### CD4 cell count increase after ART initiation

**Figure 1:**
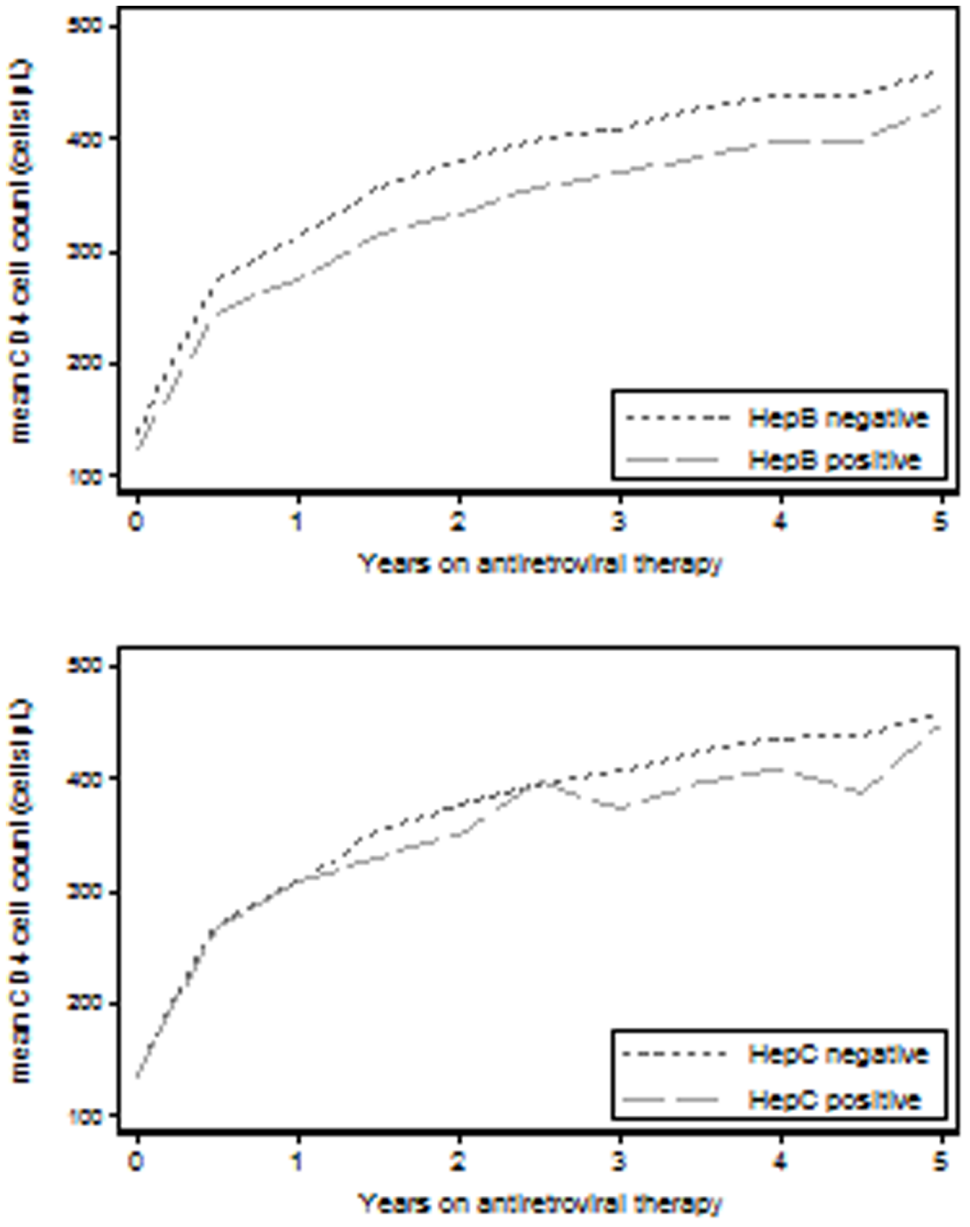
CD4 evolution after initiation of antiretroviral treatment according to co-infection with hepatitis B virus (upper graph) and hepatitis C virus (lower graph).

The mean CD4 cell count at different time points after ART initiation is shown in Figure 1. For HBV(+) individuals, a CD4 count of 275, 370 and 430 cells/μL was seen at one, three and five years after ART initiation, respectively. The corresponding values for HBV(−) individuals were 311, 409 and 461 cells/μL. In adjusted analysis, a significant lesser increase in CD4 count was estimated for HBV co-infection (Table 2). This effect was slightly more pronounced when individuals starting tenofovir during follow-up were excluded (coefficient -5.35 (95% CI −7.60;−3.10)). For HCV co-infection, the CD4 count was 308 cells/μL at one year of ART, 374 cells/μL at three years and 448 cells/μL at five years of ART. The corresponding values for HCV(−) individuals were 307, 406 and 458 cells/μL. No significant difference in CD4 count increase was found for HCV in multivariate analysis (Table 2). In time to event analysis, CD4 recovery (reaching a CD4 count of at least 350 cells/μL) was significantly delayed for HBV (adjusted hazard ratio (HR): 0.80) but not for HCV co-infection (adjusted HR: 0.90), see Table 2.

**Table 2:** Estimated effect of hepatitis B or C co-infection on CD4 count change after initiation of antiretroviral treatment.

**Mixed effects model (change in CD4 count)** a				
	Coefficient	*P*-value	Adjusted coefficient^b^	*P*-value
Hepatitis B	−4.2 (−6.1; −2.3)	<0.001	−4.9 (−6.8; −3.0)	<0.001
Hepatitis C	−1.5 (−4.3; +1.2)	0.28	−1.4 (−4.1; +1.4)	0.34
**Time to event analysis (CD4 cell count>350 cells/μL)**				
	Hazard ratio	*P*-value	Adjusted hazard ratio b	*P*-value
Hepatitis B	0.78 (0.68–0.90)	<0.001	0.80 (0.70–0.92)	0.002
Hepatitis C	0.92 (0.76–1.12)	0.39	0.90 (0.74–1.09)	0.31

a Linear mixed effects model. The coefficient assesses the effect of the covariate (hepatitis B or C) on the slope (or change) of the CD4 count (unit: cells/μL/year); 95% confidence interval given in parentheses.

b The multivariate model included the following potential confounding factors: age, gender, baseline CD4 count, baseline hemoglobin, baseline body weight, baseline WHO clinical stage, type of NNRTI or NRTI used.

### Mortality after ART initiation

**Figure 2:**
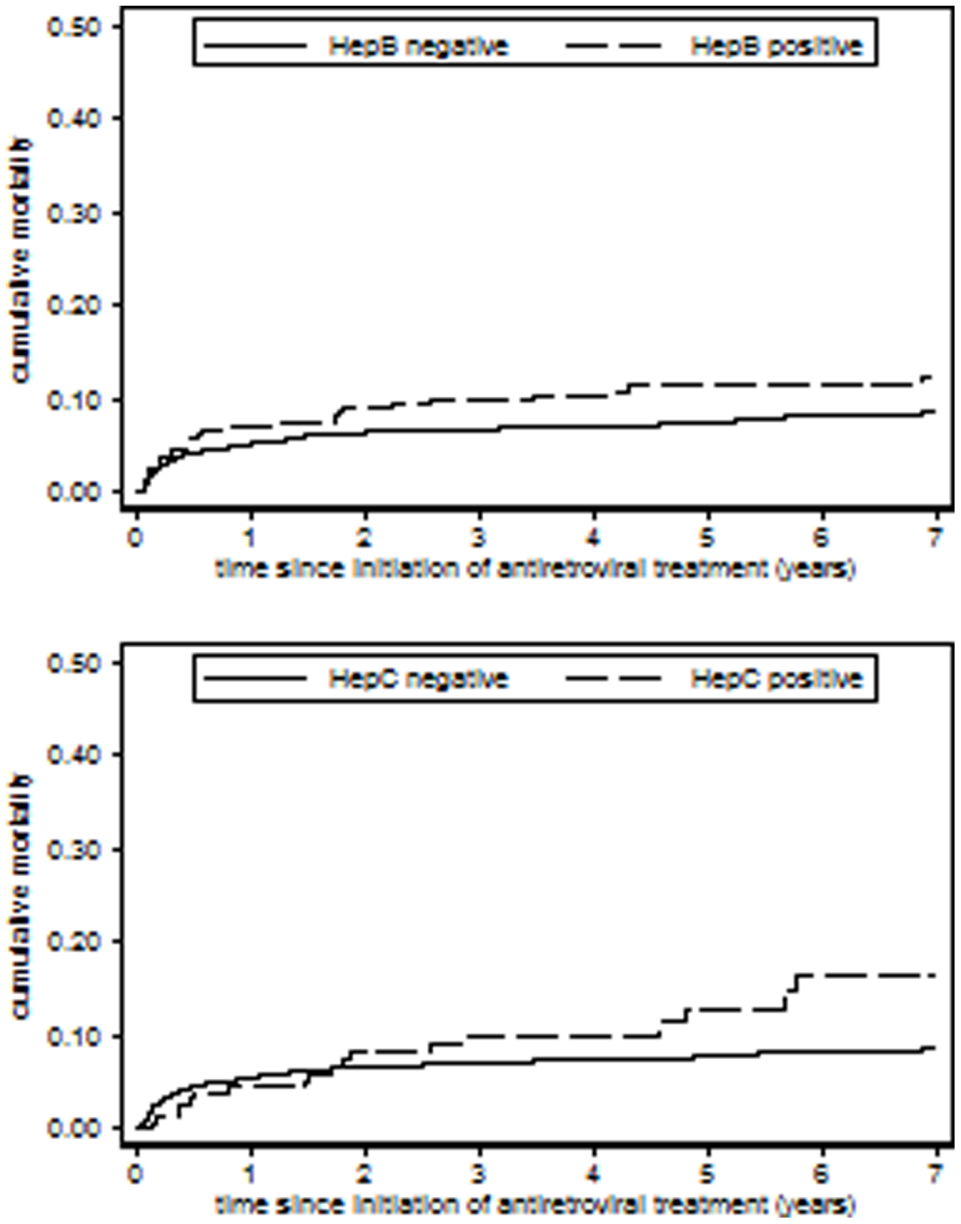
Kaplan-Meier graph showing cumulative mortality after initiation of antiretroviral treatment according to co-infection with hepatitis B virus (upper graph) and hepatitis C virus (lower graph).

A total of 240 individuals died after ART initiation, after a median of 4.2 (IQR 1.4–16) months. Figure 2 displays the cumulative incidence of mortality, stratified by hepatitis status. During the first year of ART, no statistically significant effect of HBV or HCV was observed in univariate analysis (Table 3). In adjusted analysis, a non-significant 1.4-fold increased risk of mortality was seen for HBV co-infection. After the first year of ART, the adjusted risk of mortality was 2-fold increased in HBV co-infection and 3-fold increased in HCV co-infection. Over the entire follow-up period, mortality was 60% higher in HBV co-infection in adjusted analysis (*P* = 0.010). The effect remained unchanged if time-updated CD4 count was included in the analysis (adjusted HR 1.5; 95% CI 1.1–2.2; *P* = 0.025). The effect was still observed excluding all individuals that started tenofovir during follow-up (adjusted HR 1.6; 95% CI 1.1–2.4; *P* = 0.028).

**Table 3:** Effect of hepatitis B or C co-infection on mortality at different time periods after ART initiation in Phnom Penh, Cambodia, 2003–2012 (N = 3089).

	n/N (%)	Crude HR	*P*	Adjusted HR^a^	*P*
≤**1 year of ART**					
Hepatitis B					
No	142/2748 (5.2)	1		1	
Yes	23/341 (6.7)	1.3 (0.8–2.1)	0.21	1.4 (0.9–2.1)	0.16
Hepatitis C					
No	158/2926 (5.4)	1		1	
Yes	7/163 (4.3)	0.8 (0.4–1.7)	0.52	0.91 (0.4–2.0)	0.81
**>1 year of ART**					
Hepatitis B					
No	61/2431 (2.5)	1		1	
Yes	14/296 (4.7)	1.9 (1.0–3.3)	0.034	2.0 (1.1–3.7)	0.018
Hepatitis C					
No	63/2582 (2.4)	1		1	
Yes	12/145 (8.2)	3.6 (1.9–6.8)	<0.001	3.0 (1.5–6.0)	0.002
**Overall**					
Hepatitis B					
No	203/2748 (7.4)	1		1	
Yes	37/341 (10.8)	1.5 (1.1–2.1)	0.025	1.6 (1.1–2.3)	0.010
Hepatitis C					
No	221/2926 (7.6)	-		-b	
Yes	19/163 (11.1)	-		-b	

a The multivariate Cox regression model including the following potential confounding factors: age, gender, baseline.

b Not calculated due to violation of the proportional hazard assumption.

CD4 count, baseline hemoglobin, baseline body weight, baseline WHO clinical stage.

ART: antiretroviral treatment.

### ART-related toxicity

**Figure 3:**
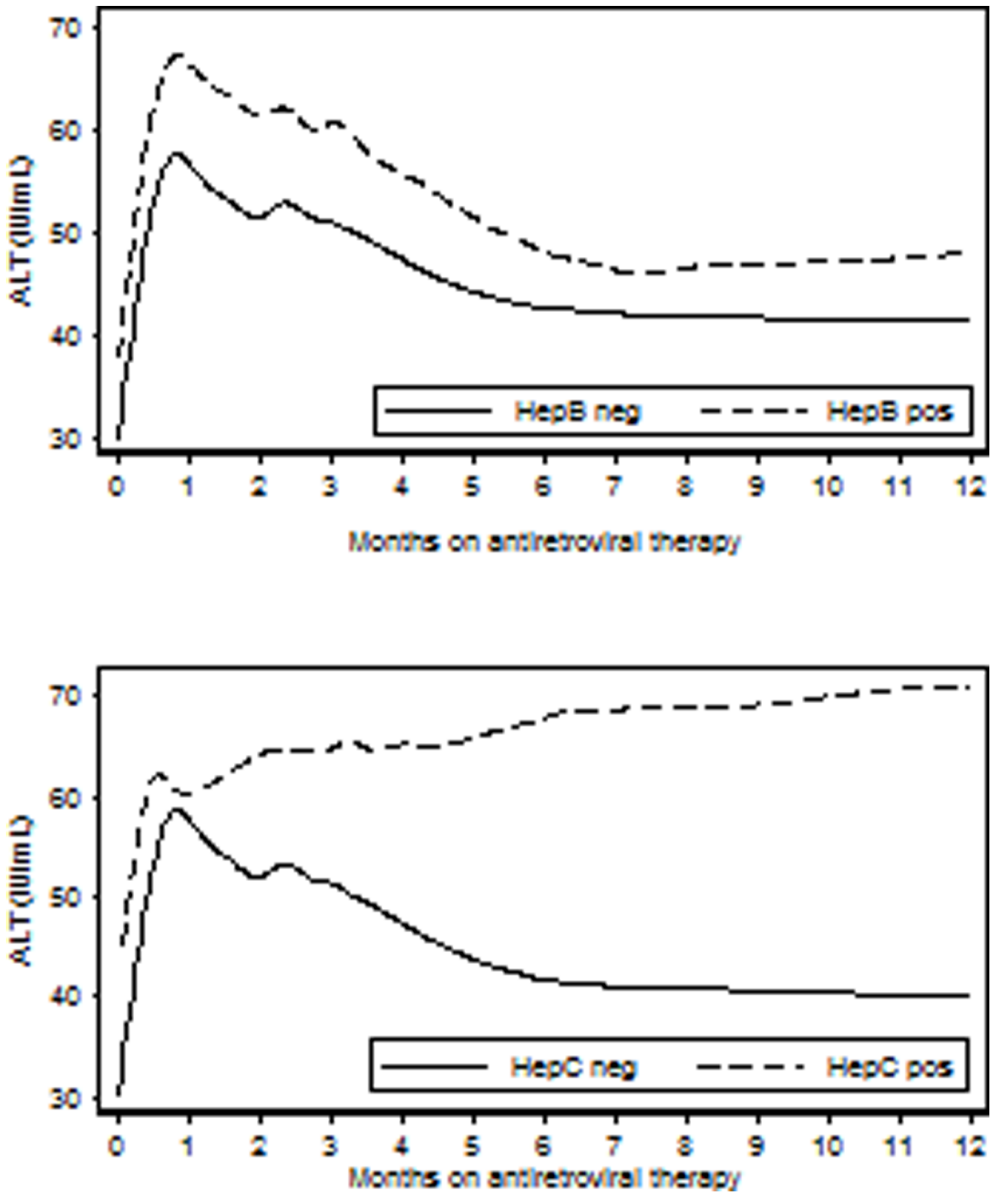
Evolution of liver function tests (ALT) after initiation of antiretroviral treatment according to Hepatitis B (upper graph) and Hepatitis C (lower graph).

A total of 180 (5.8%) individuals discontinued efavirenz or nevirapine due to severe livertoxicity. The risk was highest in co-infected patients treated with nevirapine (11.3% for HBV and 15.2% for HCV), compared to 8.0% for HBV and 6.9% for HCV co-infected individuals treated with efavirenz. Both in adjusted and unadjusted analysis, a significant increased risk of livertoxicity was seen for both HBV and HCV co-infection (Table 4). This effect was irrespective of type of NNRTI used, although nevirapine use was independently associated with a 50% increase risk of livertoxicity. No difference in mortality was observed between those with or without severe NNRTI-related toxicity. The main findings remained unchanged when excluding those individuals that had taken antituberculosis treatment while on ART. With livertoxicity of WHO grade II or more as outcome, HBV co-infection a 1.4-fold increased risk (OR 1.4; 95% CI 1.1–1.8) and HCV had a twofold increased risk (OR 2.0; 95% CI 1.4–2.8). HCV co-infection was associated with a sustained increase of ALT levels up to one year after ART initiation (Figure 3), that subsequently reversed (not shown). There were 182 individuals discontinuing ART due to skin rash, but there was no statistically significant difference for neither HBV nor HCV co-infection.

**Table 4:** Effect of hepatitis B or C co-infection on the risk for severe NNRTI-related toxicity after ART initiation in Phnom Penh, Cambodia, 2003–2012 (N = 3089).

	Livertoxicity				Skin rash			
	n/N (%)	OR^a^	aOR^a^^,^ b	*P*	n/N (%)	OR^a^	aOR^a^^,^ c	*P*
**Hepatitis B**								
No	145/2748	1	1		167/2748	1	1	
	(5.3)				(6.1)			
Yes	35/341	2.5	2.3	<0.001	16/341	0.8	.0.8	0.56
	(10.3)	(1.3–3.0)	(1.6–3.5)		(4.7)	(0.4–1.3)	(0.5–1.4)	
**Hepatitis C**								
No	160/2926	1	1		172/2926	1	1	
	(5.5)				(5.9)			
Yes	20/163	2.4	2.8	<0.001	11/163	1.1	1.2	0.59
	(12.3)	(1.5–4.0)	(1.7–4.6)		(6.7)	(0.6–2.2)	(0.6–2.3)	

a 95% confidence interval given in parenthesis.

b Adjusted for age, gender, baseline CD4 count, baseline body weight, type of NNRTI (efavirenz vs nevirapine), baseline liverfunction tests (serum alanine aminotransferase).

c Adjusted for age, gender, baseline CD4 count, type of NNRTI (efavirenz vs nevirapine).

aOR: adjusted odds ratio; NNRTI: non-nucleoside reverse transcriptase inhibitor.

## Discussion

This is one of the few studies reporting on long-term ART treatment outcomes from a program setting in a low income country in South-East Asia. Both HBV and HCV were associated with increased late mortality while on ART and increased NNRTI-related livertoxicity. The CD4 count recovery was less pronounced for both co-infections, but only statistically significant for HBV co-infection.

Strengths of the study include the relatively large sample size, long patient follow-up, the standardized patient management and the prospective data collection using standardized data collection tools. Moreover, the data originate from a programmatic setting and hence are more likely to reflect the reality on the ground. A number of important limitations need to be acknowledged. We only tested for HBV by measuring HBV surface antigen at baseline. Ideally, we should have done a full laboratory evaluation for HBV. HCV diagnosis should ideally have been confirmed by a molecular test. Causes of death, especially whether they were liver-related would have been of interest. ART adherence and HIV-1 viral load data were also not available. Although intravenous drug use was found to be relatively uncommon in our program, this information was not systematically collected in our database.

Although our findings on mortality concur with those from several African, European and American studies [24]–[28], the effect of HCV and HBV on mortality is in contrast with the few published studies conducted in Asia [18], [19], [21]. Possible reasons to consider include the longer follow-up period and/or the larger sample size in our study. Importantly, our data concur with preliminary data from a large Asian multicountry study with a long follow-up period that were recently presented [29]. The mechanism behind this increased mortality remains to be eluded. NNRTI-related drug livertoxicity is unlikely to contribute, since mortality was similar in those not displaying toxicity in our study. Differences in adherence or HIV-1 virological response could have played a role, although overall adherence has been found to be excellent in our program, with very low proportions of treatment failure irrespective of co-infection status [30], [31]. Adjusting for the difference in CD4 count response hardly altered the estimates, arguing against a major role. HCV or HBV induced liver pathology and death remains a likely contributor [32]. In a recent large European study on HCV, increased liver-associated mortality was observed despite ART use [24]. This would call for increased availability of HCV treatment in low-income countries, as has been called for by several international organisations. The recent WHO recommendation to include tenofovir in first line ART regimens would also be expected to better control HBV and reduce the emergence of lamivudine drug-resistant HBV [33], besides lowering the chance of hepatic steatosis associated with stavudine and zidovudine [34]. Interestingly, a recent study from Africa reported that the increased mortality in HBV co-infection in their program was thwarted once HBV co-infected patients systematically received tenofovir-based first line ART [35].

In contrast with HBV, no significant effect of HCV on CD4 recovery was seen in our study. Although this might be a genuine difference between HCV and HBV, other possibilities should be considered including the smaller number of patients with HCV and the possible inclusion of false-positive and resolved cases of HCV in our study. At the global level, several studies found an effect of HCV or HBV on CD4 recovery including a meta-analysis [35]–[42], while others did not [11], [28], [43]–[48]. Differences in local epidemiology and prevalent genotypes, follow-up time and population characteristics (in particular the relative importance of intravenous drug users) might have contributed to this [40], [41], [45]. The majority of African and Asian studies did however report a lesser CD4 increase with HCV or HBV co-infection, but the difference did not reach statistical significance in several of them [18], [19], [21], [22], [35], [41], [46], [47]. Most likely, the effect of HBV or HCV on CD4 cell recovery, if any, is not large and hence could easily be missed in underpowered studies or with short follow-up. In line with this, reduced CD4 recovery was recently reported for HBV and HCV in the large Asian multicountry study mentioned above, and for HCV in a recent meta-analysis [29]. The clinical significance of any such effect in terms of AIDS-related events also remains to be determined. Possibly, it could be an indicator of ongoing inflammation, which has been implicated in enhanced progression of liver fibrosis but also to a range of long-term complications including increased cardiovascular disease and cancer [49]–[52]. Whether the reduced CD4 recovery would warrant earlier ART initiation in co-infected patients, as currently recommended in high income countries, requires further study. Early ART initiation might also contribute to slower liver fibrosis progression [15], [16].

The increase in NNRTI-related toxicity is in line with most other reports from the region [19], [21], [53]. The fact that livertoxicity was clearly higher with the use of nevirapine argues for the systematic use of efavirenz for co-infected patients. A prolonged increase in ALT levels after ART initiation was seen in HCV co-infection. Interestingly, in an American study, ART initiation was found to be associated with increased HCV viral load levels, accompanied by a prolonged hepatic flare [54].

Our findings raise a number of important public health questions. First, related to HBV, the question remains whether effective suppression of HBV – eg with combination therapy – might improve overall patient outcomes [10], [55]. Although the increasing use of tenofovir in first line ART regimens in low and middle income countries would be expected to yield more effective HBV control, monitoring strategies that can be realistically implemented in resource constrained settings should be in place as well. Since HBV is a non-curable infection, a long-term management strategy will be required. In contrast, HCV is a curable infection. Importantly, several studies have shown that HCV treatment in low and middle income countries is feasible and effective [56], and there are indications that treatment outcomes with standard treatment are better in Asia [57], [58]. The development of interferon-free regimens, relying on directly acting agents, is also an exciting recent evolution [59]. While many barriers remain in terms of cost, access to treatment and availability of the appropriate laboratory monitoring tools, simplified and adapted treatment strategies should be explored. The story of the global ART scaling-up exemplifies that concerted international (advocacy) efforts can yield dramatic price reductions and improve access to and quality of care in resource constrained settings [60].

A number of topics remain to be addressed in future studies. This includes detailed, prospective studies on the extent and evolution of liver disease in co-infected individuals, as well as the impact of hepatitis treatment and ART on liver disease and the associated mortality. Epidemiological studies would be instrumental to better understand the risk factors and mechanism of infection, and the prevailing genotypes. This would also provide avenues for preventive efforts. Qualitative studies might provide useful insights into the psychosocial context of HCV co-infection, intravenous drug use and other factors possibly contributing to their worse prognosis, which might be valuable to improve patient care. Finally, operational research will be required to identify optimal care models for HBV and HCV co-infection [61].

In conclusion, HBV and HCV co-infection was common in this ART program in Cambodia. Co-infection was associated with increased liver-related ART toxicity. Despite fairly similar CD4 count recovery, HBC-infection was associated with a 3-fold increase in long-term mortality. With regards to HBV co-infection, CD4 count recovery was less pronounced, and a 60% increase in mortality was observed throughout the follow-up period. Further studies on the cause of death and extent of liver fibrosis in hepatitis co-infection are required, and the effect of early ART initiation and providing effective treatment for hepatitis co-infection on should be explored.
